# Hemorrhoids and Treatment

**Published:** 1895-04

**Authors:** C. T. Young

**Affiliations:** Waco, Texas


Texas Medical Journal.

ESTABLISHED JULY, 1885.

Published Monthly.—Subscription $2.00 a Year.

Vol. X. AUSTIN, APRIL, 1895. No. 10.

### Original Contributions.

For Texas Medical Journal.

### HEMORRHOIDS, AND TREATMENT.

BY C. T. YOUNG, M. D., WACO, TEXAS.

Read before the Waco Medical Association, February 12, 1895.

FOR convenience I will divide hemorrhoids into the old division, external and internal. External hemorrhoids are those developing outside the external sphincter muscle, due generally to a varicosity of the external hemorrhoidal vein, and is therefore an affection of the general venous circulation. Internal hemorrhoids are those developing inside the external sphincter muscle. They develop from the internal or superior hemorrhoidal veins, and are connected with the portal circulation.

External hemorrhoids, thrombus, varicose, inflammatory connective tissue. Thrombotic pile is a small round or oblong tumor, due to coagulation of blood in a dilated hemorrhoidal vein, or rupture of the vein, with consequent extravasation of blood into the subcutaneous connective tissue of the anus. They are painful at first, owing to unusual tension, but if left alone, for ten days or so, the pain generally subsides, and the clot is usually absorbed, leaving only a slight induration; if irritated however, these piles may set up a cellular inflammation, which will eventuate in marginal abscess or subtegumentary fistula.

*Treatment:* Both cold and hot applications have been highly recommended, but the radical treatment is so sure and simple, I

think is the only one due consideration. Having cocainized the parts, lay the sac freely open, clean out the cavity well, and wash with boracic acid solution, then pack with iodoform gauze; if necessary apply slight pressure until bleeding is controlled. After twenty-four hours remove packing and allow wound to heal, keeping parts clean by bathing two or three times a day in cold water.

VARICOSE HEMORRHOIDS.—This form of external piles are tumors composed of varicose veins about the verge of the anus. They are seen more frequently in men of sedentary habits, and women in the higher classes of society. They are usually due to constipation and prolonged straining at stool. These tumors are not circumscribed, as are the last, and it is difficult to locate their boundaries. During and after the acts of defecation, or straining, they swell and become very prominent, and cause an uneasiness rather than pain, which disappears after the circulation is re-established.

*Treatment:* When uncomplicated these piles should be treated with a sponge frequently dipped in ice-water, for about fifteen minutes at each sitting, two or three times a day, and at bedtime apply thoroughly an astringent ointment. Dr. Tuttle, of New York, uses ung. stramon. ung. belladona, ung. ac. tannic aa 1 part. I think any good astringent ointment will suffice. The patient's bowels should be regulated, and he should avoid prolonged straining.

INFLAMMATORY EXTERNAL HEMORRHOIDS. — The tumors originate in the inflammation and swelling of the anal folds. They are called Cripps' œdematous piles. They are tumors having a muco-cutaneous covering containing distended arteries and veins with more or less serous effusion. They are due to traumatism, or irritation either from within or without the rectum. Anal or rectal ulcers with irritating discharges, fissures, chancroids, improper toilet material, rough wiping, improper use of the enema tube, rectal masturbation, hard fœcal masses, etc., may all produce them. This variety of piles being so very painful, disables more than any other kind.

*Treatment:* The cause of these piles should be removed, then cold water with sponge described above will cure very readily.

CONNECTIVE TISSUE HEMORRHOIDS.—This class of piles is often improperly spoken of as condyloma, (so says Kelsey). They are truly connective tissue piles having a mixed cutaneous and muco-cutaneous covering. They are due to irritation causing hyperæmia of the parts. That may result from either of the three varieties mentioned above, or malignant or specific disease of the rectum. They are liable to become inflamed from very slight irritation or traumatism of the parts, and get very painful, which sometimes leads to ulceration and sloughing.

*Treatment:* They should be treated by regulating the bowels, frequent use of cold water, and if necessary clipped off with scissors.

INTERNAL HEMORRHOIDS.—While there has been long division of internal hemorrhoids it is only necessary to make two classes; that is, capillary and venous or varicose.

Capillary hemorrhoids are composed of the terminal branches of the superior hemorrhoidal arteries and veins, and their connecting capillaries. They are situated upon the rectal mucous membrane, with a very thin covering, which accounts for their bleeding so easily when touched. The tumors are very small and can not be made out by touch, but come into view when the patient strains, and rectum is opened by surgeon's thumbs, or speculum. I have never treated a case of this class of piles, but have seen both pure nitric acid and electro-cautery used with good results in each case.

Venous, or varicose internal hemorrhoids, are recognized as the ordinary internal piles; they are a mass of freely anastomosing veins, bound together by connective tissue. The veins are tortuous, usually varicosed and dilated into sacs and pouches. Many of this class of cases are due to constitutional and local causes, which, if properly treated, the piles will disappear. Intestinal indigestion, hepatic congestion, proctitis, cystitis, urethral stricture, retroversion or prolapse of uterus, rectal ulceration, kidney disease, heart disease, are frequent causes of hemorrhoids. By treating correctly any of these troubles, with soothing applications to the hemorrhoids, frequently the hemorrhoid disappears. When these measures fail, an operation is imperative. I will state, before starting in to discuss operating, that patients should all be prepared for the operation, and the operation done under an anæsthetic. I shall only mention five operations, viz.: injection, clamp and cautery, ligature, crushing and Whitehead's operation. The injection method is very successful in simple internal varicose hemorrhoids where there are no complications, and the sphincters are comparatively relaxed. It requires some skill and experience to apply it. Its aim should be to produce inflammatory induration in the hemorrhoid and subsequent absorption, and not to produce sloughing. The tumor should be brought into view with as little manipulating of parts as possible, thoroughly cleaned, the needle having been previously cleaned, is introduced from its base into its center. The blood should then be pressed out (as much as possible), and about four minims of the fluid injected—I use the solution recommended by Dr. Tuttle. It is a modified Schuford's. Ac. carb., ʒiss; ac. salicyl, ʒss; soda biborate, ʒi; glycerine, q. s. ad., ℥i.

This treatment can be used without taking the patient from his daily vocation, and gives no pain.

Clamp and Cautery.—When there is an operation imperative, I think none equal to the clamp and cautery. The tumors should be located and counted before the sphincters are dilated. The cutaneous portion of the hemorrhoid should be dissected off with scissors, then the tumor should be grasped at the base by the clamp, in a line parallel with the axis of the gut. Cut it off with scissors, and cauterize the stump. Dust the parts with iodoform, insert a small piece of iodoform gauze, apply a good compress and a snug T bandage. On the third day move bowels; in one week or less your patient will be attending to his duties.

Ligature.—You are all familiar with Allingham's ligature operation, 'tis unnecessary to give the technique. I will simply point out its disadvantages (to my mind) compared to the clamp and cautery. First, it takes longer to perform the ligature operation; second, there is more pain after operation from sphincteric contraction, as a patient very well described to me on one occasion: "I could get along very well if I did not have so much of this d—d winking." Third, instead of having a smooth, healthy ulcer the day we operate, we have no ulcer till the ligature sloughs off; fourth, the operation usually necessitates the use of the catheter; fifth, it involves the danger of secondary hemorrhage; sixth, it confines the patient to bed for a considerably longer time.

Crushing.—This is virtually the clamp operation without the cautery. It is perhaps less painful than clamp and cautery, but it is not near so safe in regard to hemorrhage and sepsis. Its usefulness is greatly limited, as there are but few cases applicable to this operation.

Excision, or Whitehead's Operation.—This operation consists in removing the whole pile-bearing area. An incision is made around the anus at the junction of the skin with the mucous membrane. The latter, with the hemorrhoidal tumors, is dissected from the muscular wall and upward till the upper limit of the hemorrhoids is passed, then excised. The mucous membrane is then brought down and stitched to the skin. When we get union by first intention, and have not cut too far into the skin, this operation is an ideal one, so far as result is concerned. If there should be a slight mistake made in operating, you will have stricture (which is worse than the hemorrhoid) ulceration or prolapse, and unless a man is an expert surgeon he had better select some other operation for hemorrhoids. I have been there.

### DISCUSSION.

Dr. Halbert:—Mr. President and gentlemen: I was very much interested in the paper. I have never tried Whitehead's operation. I have seen it performed several times, and there are two or three points in it that impressed me. I remember the last time I saw it performed. The first thought that struck me was that if I performed that operation I would dissect out that fellow's external sphincter muscle. The next difficulty in the way was the fear that we could not sew together the mucous membrane closely and nicely and snugly like it ought to be done. He just dissected off a little of the mucous membrane and then made his attachments, and then, as he cut off more of the mucous membrane, he attached it again. I don't believe, on the whole that Whitehead's operation is justifiable, except in very bad cases, where there is prolapsis of the gut. But, as the doctor says, when it is properly performed and the result is proper, it is an ideal operation. I think the best operation, on a general basis, is the operation with a clamp and cautery. It is simple and rapid, and the patient recovers very rapidly. The operation with the ligature he speaks of, I had a case of that kind once, and it nearly scared me to death. He bled himself nearly to death before I got to him. Before I left him his pulse was a little too quick, but I thought it was nervousness. I know that my trouble was that instead of applying the ligature at right angles with the gut, I must have applied it parallel with the gut.

Dr. Foscue:—I saw Dr. Shuford operate several times, recently, in which he used his speculum and his fluid. Don't know what the result is going to be yet. It is a very nice operation. He doesn't use any anesthetic, uses cocaine only, but gives you pain, although he claims that he doesn't give a particle of pain. I think, in the majority of those cases the operation can be done under cocaine, that is, external hemorrhoids.

Dr. Black:—I recommend the treatment as set forth and can add nothing to it. The paper presents the subject in all forms. I have always used the ligature in the few cases I have treated, except those little bluish hemorrhoidal tumors that are external and cause so much itching and pain. I have not tried any method except the ligature, and my method has been to transfix the tumor and ligate it just as you would ligate the pedicle in ovariotomy. I have not treated many cases, but I have not failed. I do not think that my experience is worth drawing on, to teach any one here. I do not know that I would follow the same plan that I have always followed. It occurs to me, that a man who is a surgeon, must be governed by the condition of the tumors and the character of the tumors. I have never been much impressed with the idea of using the injection. I know that it succeeds, but I know that it sometimes produces horrible ulcers and fistules and abscesses. I have never used it. It has never occurred in my practice, but it has in other gentlemen's practice, and I have seen accomplished surgeons refuse to operate in that way after they had tried it once or twice, saying that they believed it was dangerous, and while it may succeed eight times in ten, if it fails twice in ten, I do not think it should be used. I prefer the ligature. I do not know that I have any reason to prefer that over the clamp and cautery, but I have used the ligature and I have not used the clamp and cautery, although I have seen it used half a dozen times, probably, and with success. I have been profited by what has been said about the matter; am glad to have heard Dr. Young's paper. I should be glad to hear this subject well discussed for my own benefit. It is an important subject.

Dr. Hunter:--Just a word, Mr. President. I am very much pleased with the doctor's paper. It is a great paper, and it shows that he is thoroughly conversant with the subject and with the technic of the different operations. Different surgeons have had different results from the different operations. The surgeon who is fond of the knife, never agrees to the injection operation. Now. I think, that the injection operation, take it altogether, causes less pain than any other operation. The patients, as a rule, in my judgment, get well quicker than with most any other operation. They don't have to stay in bed as long. If there are a good many piles, as a matter of course it is not best to operate on all at once in these cases. Take one at a time and the man will hardly have to go to bed at all in a great many cases. The other operations are good operations. I believe I prefer the clamp and cautery. But the ligature is an ideal operation with a great many. But take it altogether, I think, I like the injection operation best.

Dr. Olive:—I think, Mr. President, the gentleman has dealt pretty thoroughly with the pathology of the subject, and his paper certainly deserves pretty thorough consideration at the hands of the members present, and as far as pathology is concerned, I don't think anything can be added. It shows a great deal of research, and he has dealt with the subject in a masterly manner. As to the treatment, I don't believe that he mentioned that the bowels should be thoroughly opened, but it was simply an oversight if he didn't, before any sort of operation is resorted to. As to Whitehead's operation, I think it is one that can only be resorted to as a dernier ressort and by a surgeon of great skill in that class of operations, and I think it requires a great deal of care and skill in doing it correctly and deriving any benefit from the operation. I think it a good operation, though, when it is used as a dernier ressort. I agree with not only the essayist, but also with Dr. Halbert as to the clamp and cautery. I think that is an ideal way of dealing with internal hemorrhoids, and I think it is a very useful method, and one that is not used as often as it should be.

As to the injection method, I think that is a method that is not to be resorted to in an indiscriminate and unscientific manner. I think, that as the essayist spoke of it, it is certainly a useful remedy.

I think a distinguished specialist in a town about the size of Waco, a year or two ago, had a case that he injected into the bowels some sort of fluid, I don't know what, and it seems to me that the result of that was an abscess of the liver, and I think patient died with the piles, or the result of the piles treated by the injection method.

Dr. Gawne:—I would like to ask Doctor Young if this Whitehead's operation is the same operation that Pratt has been making such a furor about in Chicago?

Dr. Young:—Yes, sir.

Dr. Gawne:—I do not know that I have had experience enough in the management of piles to be of any importance to any one, though I have made a number of operations. My method has generally been with the needle and ligature. I usually dilate the sphincter thoroughly so as to get at the piles readily. Some of these piles are almost fibrous in feeling. These I have always *ligated*, and some of the smaller ones I have injected, with very good success, as I have thought. But I have seen a great deal of the results of Pratt's operation. This man Pratt, of Chicago, is quite a homeopathic gentleman. I have not seen the operation as done by the doctor himself, but as done by his satellites that took up the practice in my immediate neighborhood who advocated the Pratt operation, or amputation. as they called it, of the hemorrhoidal area, for almost every ailment of the human system. And I know of a case that was actually operated on for a skin affection of the face. Another case of hopeless insanity. Those were both patients of mine, but they went over to these people, because they promised to cure them. I saw, subsequently, some very bad results from this operation. I am not claiming that it was on account of the operation in itself, but it appeared to me that it was on account of the operation being perhaps poorly done. In many of these cases the patient would be troubled afterwards by an ulceration at the point where the skin and the mucous membrane were stitched together, and would suffer a great deal. I know some of these people that had been patients of mine and had gone over to this wonderful doctor, who cured all things by amputating the hemorrhoidal area. I, perhaps, would ask them how they were getting along. Well, they would say, doctor, I am getting along pretty well, but I can't sit down. They hoped they would be better, but they couldn't sit down. And some of these cases came subsequently under my observation, and I found there to be a line of ulceration around the point where I suppose the stitches had been put in binding the mucous membrane and the skin together. I think myself that that would be a valuable operation under certain circumstances, but it has got into very bad repute in my part of the country on account of its being advocated for all sorts of ailments.

Dr. Compton:—Mr. President, I agree with the gentleman, except my plan in hemorrhoids is ligature. I have used the injection, but I have gotten better results out of the ligature, and for that reason I prefer it. I have enjoyed the doctor's paper very much.

Dr. King:—Mr. President and gentlemen: I do not know that

I can add anything to the paper and to what has been said already on the subject. I want to thank the doctor for the paper. I think it is a very complete, concise paper on the subject, and one that we all appreciate and enjoy.

DR. HALE:—Gentlemen: I have enjoyed the paper very much indeed. It has been a paper of the kind I like to hear. It is practical and to the point. As to the different methods, they have all been used in the hands of different men successfully to a greater or less extent, and they all have their advantages. To give my experience wouldn't be worth anything to anybody, but I want to thank Dr. Young for the paper he has presented us.

DR. YOUNG:—Gentlemen: I think Dr. Halbert is jesting a little about being afraid he would dissect out the muscle, because he knows very well that he could get the mucous membrane down without dissecting out the muscle. As to Dr. Halbert's case with that ligature, that was simply due to the weakness of the tissues on one side of the ligature. One side broke down much more readily than the other. Those cases Dr. Black mentioned, if he had applied a little ice water to them they would have gone back anyway.

Dr. Kelsey was one of the finest hemorrhoidal surgeons in the United States. He has tried this injection method very thoroughly. He has injected carbolic acid from 10% clear on up to pure acid, and has never gotten very favorable results. There is a way to inject this. You have got to empty that pile and get it clear of blood, and get a perfectly clean needle, and inject a fluid that will not set up inflammation.

Dr. Olive spoke of the bowels. I mentioned, before I started out with the internal piles, that the patient was to be properly treated. I never went into that because I knew every one knew how that was.

Where there are no ulcerations, or any complications and things of that kind, and the sphincters are moderately dilated, as they usually are with these hemorrhoids, you will get good result from the injection, provided you do not use it strong.

Dr. Gawne speaks of Pratt's operation. I saw a party come over into New York to Dr. Kelsey to be operated on after Pratt had performed an operation, and the trouble with that operation was that he had extensive prolapsation, because he had cut too far into the skin and it contracts and pulls the mucous membrane down.

Dr. Compton speaks of the ligature in preference to the injection. Well, I think, if he gets the proper cases, he would like the injection if he would try it. I have seen that used several times, and I have used it myself, but then you have to have the proper cases. You have not got to use it when there is any complication, nor when the sphincters are dilated, or anything of that kind.

N. A. OLIVE, Secretary.

